# Association between aesthetic satisfaction and chronic postsurgical pain in breast cancer patients treated with one stage prosthesis implantation

**DOI:** 10.1038/s41598-022-05185-z

**Published:** 2022-01-24

**Authors:** Baona Wang, Peng Gao, Jing Wang, Hui Zheng

**Affiliations:** 1 Department of Anesthesiology, National Cancer Center/National Clinical Research Center for Cancer/Cancer Hospital, Chinese Academy of Medical Sciences and Peking Union Medical College, Beijing, 100021 China; 2 Department of Breast Surgical Oncology, National Cancer Center/National Clinical Research Center for Cancer/Cancer Hospital, Chinese Academy of Medical Sciences and Peking Union Medical College, Beijing, 100021 China

**Keywords:** Cancer, Signs and symptoms

## Abstract

This study retrospectively studied the incidence of chronic post-surgical pain (CPSP) following single-stage implant-based breast reconstruction (IBBR) and evaluated the possible risk factors. This was a retrospective cohort study, involving all patients undergoing single-stage IBBR between January and December 2019. The follow-up was completed between January and March 2021. The scores for satisfaction (SS) were based on the BREAST-Q, while the pain burden index (PBI) was used to assess the degree of CPSP. The questionnaires were completed by 159 patients. CPSP occurred in 48.43% of the patients, 2.52% of them being severe cases. Significant predictors for the development of CPSP in the univariate analysis included severe acute postoperative pain (PP), a history of preoperative chronic pain, psychological disorders, SS with the reconstructed breasts, and whether there were any regrets about having had the reconstruction. Multivariate analysis identified severe acute PP (odds ratio (OR) = 2.80, 95% confidence interval (CI) = 1.16–6.79, p = 0.023), a history of preoperative chronic pain (OR = 3.39, 95% CI = 1.42–8.10, p = 0.006), and the SS (OR = 0.86, 95% CI = 0.75–0.99, p = 0.034) as being independently associated with the development of CPSP. In subgroup analysis, the PBI of the patients in the SS < 12 group (p < 0.001), the bilateral group (p < 0.01), and the severe acute PP group (p < 0.005) was significantly higher than the PBI of those in the control groups. This study demonstrated a significant incidence of CPSP following single-stage IBBR, and the patients with lower SS of their reconstructed breasts developed more CPSP. Lower SS, bilateral procedures, and severe acute PP were predictors of higher PBI.

**Trial registration:** Registered in Chictr.org.cn registry system on 24 February 2020 (ChiCTR2000030139).

## Introduction

Breast cancer is the most commonly diagnosed form of cancer among women in China, affecting 304,000 patients per year^1^, although there has been a decrease in mortality and an increase in 5-year survival rates due to recent advances in treatment. With the increase in survivorship, more attention is now being paid to optimizing the quality of life. In line with this, surgical procedures have become less aggressive, with the move from radical mastectomy to modified radical mastectomy, to simple mastectomy, and to breast-conserving surgery, and from axillary lymph node dissection (ALND) to sentinel lymph node biopsy (SLNB), without impairment of the therapeutic effect. Today, direct-to-implant reconstruction (single-stage) following a mastectomy offers another choice to patients who do not have the opportunity to have breast-conserving surgery or are unwilling to conserve the breast due to the necessity of undergoing radiotherapy or the slightly higher incidence of local relapse. However, although women with breast cancer now have more opportunities to maintain their appearance, for many patients, intractable chronic post-surgical pain (CPSP) negatively impacts their quality of life.

CPSP is reported to be one of the most serious problems for patients who have survived breast cancer. The pathogenesis of CPSP is complicated and needs elucidation, and no effective treatment methods are currently available. Post-mastectomy pain has been estimated to affect 20–50% of patients^2^. In our previous study, we found that the rate of chronic pain after mastectomy is about 40–50%^3^. It was reported that for patients receiving single-stage implant-based breast reconstruction (IBBR), no more incidence of CPSP was found^4^. However, studies on CPSP in patients with single-stage IBBR have often had a very small sample size, and there has been no separate analysis of patients who received a tissue expander and those who had an implant. Apparently, it is not precise to calculate the incidence of CPSP of these two different surgical procedures together.

There is a surgical team at the Cancer Hospital of the Chinese Academy of Medical Sciences, Beijing that specializes in single-stage IBBR for women with breast cancer. Owing to the depth of their experience in single-stage IBBR and the large sample size, it was decided to retrospectively study the incidence of CPSP following single-stage IBBR and evaluate the possible risk factors, including the size of implant, postoperative complications, whether a biological matrix was used, whether patients regretted having the IBBR, and their scores for satisfaction (SS) with the reconstructed breasts, all of which have been rarely mentioned in previous studies.

## Methods

This was a retrospective cohort study. All patients who underwent single-stage IBBR at the Cancer Hospital of the Chinese Academy of Medical Sciences, Beijing, between January and December 2019 were included. The female patients, aged between 18 and 60, were identified for inclusion in the study and attended follow-ups between January and March 2021. This study was approved by the Ethics Committee of National Cancer Center/Cancer Hospital, Chinese Academy of Medical Sciences. Electronic medical records were reviewed, and the patients’ details were collected including age, body mass index (BMI), menstrual status, history of psychological disorders, history of preoperative chronic pain, the severity of postoperative pain (PP), type of surgery, whether a biological matrix was used, the size of implant, whether they had unilateral or bilateral single-stage IBBR, axillary interventions including SLNB and ALND, and the duration of surgery and anesthesia. The incidence of adjuvant therapy, namely radiation therapy, chemotherapy, and endocrinotherapy, was also documented.

All the surgical procedures were performed by the same surgical team using the same standardized techniques. All patients received IBBR right after mastectomy, and biological matrixes were used in some of them. The technique for single-stage IBBR with a biological matrix was to release the inferior origin of the pectoralis major muscle and create a subpectoral pocket. The biological matrix, which was tailored according to the individual case, was fixed to the chest wall to cover and support the lower and lateral areas of the implant and was used to completely close the pocket due to the insufficiency of the pectoralis major muscle. The implant and biological matrix were infiltrated with an antibiotic solution for at least 10 min (100 mL normal saline solution containing 40 mg gentamicin and 0.5 mg adrenaline) before the submuscular IBBR.

Standard general anesthesia was induced using sufentanil 0.3–0.6 μg/kg, propofol 1–2 mg/kg and cisatracurium 0.2–0.4 mg/kg. After laryngeal mask airway insertion, the patients were mechanically ventilated to maintain the end-tidal carbon dioxide concentration at 35–45 mmHg with a fresh gas flow of 2 L/min 60% oxygen. Anesthesia was maintained by constant inhalation of 1.5–2.5% sevoflurane and a constant infusion of remifentanil at a rate of 0.1–0.2 μg/kg/min. Sufentanil 0.1 μg/kg was added intraoperatively as required. At the end of surgery all patients received 100 mg of flurbiprofen. For patients whose visual analogue scale (VAS) was ≥ 4 within 48 h after the operation, the same dose of flurbiprofen was repeated.

CPSP was defined as: (1) pain developing or increasing in intensity after a surgical procedure. (2) Pain duration of at least 3–6 months. (3) Pain is either a continuation of acute post-surgery pain or develops after an asymptomatic period. (4) The pain is either localized to the surgical field, projected to the innervation territory of a nerve situated in the surgical field, or referred to a dermatome. (5) Other causes of pain should be excluded, e.g., infection or continuing malignancy^5^. The follow-up survey collected data concerning the development of CPSP, including its onset, location, frequency, and intensity, as well as therapeutic interventions and postoperative complications. All patients attended follow-ups between January and March 2021. They completed questionnaires about breast satisfaction and determined CPSP and pain burden index (PBI) at the same time intervals.

The satisfaction that patients felt about their reconstructed breasts was evaluated using the BREAST-Q (BREAST-QTM-BREAST CANCER CORE SCALE VERSION 2.0.) The satisfaction scale used four questions: How do your breasts look when you are dressed? Do you feel comfortable in your bra? Can you wear tight-fitting clothes? How do your breasts look when you are naked? The highest SS was 16, while the lowest was 4, and the higher the score, the greater the level of satisfaction. Although the physical well-being section in the BREAST-Q contains items concerning pain or other uncomfortable feelings, it cannot be used to evaluate pain intensity. Therefore, in this study, the CPSP data were collected using the breast cancer pain questionnaire first developed by Gartner et al.^6^, and the questionnaires were completed online or by phone. PBI was calculated using data collected in the questionnaire. It was calculated by adding the pain severity scale (0–10) from the anatomic locations of the breast, axilla, chest wall, and arm, and multiplied by the frequency of pain at each site (constantly = 5 points, daily = 4 points, occasionally = 3 points, weekly = 2 points, monthly = 1 point, and never = 0 points). The VAS level of immediate PP has been recorded carefully by a nurse from the Department of Anesthesiology. VAS ≥ 7 was defined as severe PP. Psychological disorders, including emotional distress, depression, anxiety, and anger, were assessed using short-form instruments from the Patient-Reported Outcomes Measurement Information System (PROMIS). Alcohol excess was defined as drinking more than 100 g of alcohol/day longer than 3 years.

Statistical analysis was performed using SPSS version 21.0. Data were presented as mean ± standard deviation. Risk factors for the development of CPSP were compared using logistic regression. Multivariate logistic regression was performed for the selected variables. Odds ratios (ORs) were reported with a 95% confidence interval (95% CI). As the highest SS was 16, and the patients whose SS was ≥ 12 were ordinarily more satisfied with their breasts than those with SS < 12, we divided the patients into the SS ≥ 12 vs. SS < 12 groups to compare their PBIs. Besides, the PBIs of the other subgroups, including the unilateral vs bilateral groups, the severe acute PP vs non-severe acute PP groups, and the history of chronic pain vs no history of chronic pain groups, were also compared using a Mann–Whitney U test. A significance of p < 0.05 was used.

### Ethical approval

This article does not contain any studies with animals performed by any of the authors. All procedures performed in studies involving human participants were in accordance with the ethical standards of the institutional and/or national research committee and with the 1964 Helsinki declaration and its later amendments or comparable ethical standards.

### Informed consent

Informed consent was obtained from all individual participants included in the study.

## Results

The study involved 182 patients who underwent single-stage IBBR between January and December 2019. One patient, who died from metastasis, was excluded from the final analysis. The questionnaires were completed by 159 patients. The baseline characteristics of the participants are summarized in Table 1. The median follow-up time was 17 months (range 13–25 months). Of the 159 cases analyzed, there were 2 cases of metastasis, 2 cases of local relapse, and 13 cases of postoperative complications, including 3 cases of nipple necrosis, 8 cases of implant retraction due to infection, and 2 cases of implant displacement. All the patients received single-stage IBBR after a mastectomy plus SNLB or ALND, and 9 of them received bilateral single-stage IBBR plus a mastectomy.

**Table 1:** Baseline characteristics.

Patient characteristics	Mean or frequency
Age (mean)	40.94 ± 7.12
BMI (mean)	22.13 ± 2.90
Smoker	4 (2.52%)
Alcohol excess	6 (3.77%)
**Dysmenorrhea**	
No	120 (75.47%)
Yes	25 (15.72%)
Menopause	14 (8.81%)
**Psychological disorders**	
Yes	76 (47.80%)
No	83 (52.20%)
**History of preoperative chronic pain**	
Yes	35 (22.01%)
No	124 (77.99%)
**Type of surgery**	
Mastectomy + SLNB + IBBR	109 (68.55%)
Mastectomy + ALND + IBBR	41 (25.79%)
Bilateral mastectomy + IBBR	9 (5.66%)
**Adjuvant therapy**	
Radiotherapy	35 (22.01%)
Chemotherapy	99 (62.26%)
Endocrinotherapy	58 (36.48%)
Surgery time (h) (mean)	2.21 ± 0.71
Anesthesia time (h) (mean)	2.73 ± 0.75

*BMI* body mass index, *ALND* axillary lymph node dissection, *SNLB* sentinel lymph node biopsy, *IBBR* implant-based breast reconstruction.

CPSP occurred in 48.43% of the patients in this cohort (Table 2). The amount of pain at different locations, the different PBI levels, and the VAS scores are also shown in Table 2. There were 2.52% severe cases of CPSP (VAS ≥ 7) in this study.

**Table 2:** The incidence of CPSP, the amount of pain originating from different locations, and the different PBI levels and VAS scores.

The total incidence of CPSP	48.43% (77/159)
**Location (n, %)**	
Breast	48 (30.19%)
Chest wall	34 (21.38%)
Axillary	26 (16.35%)
Arm	11 (6.92%)
**Intensity (n, %)**	
VAS 0	82 (51.57%)
VAS 1–3	37 (23.27%)
VAS 4–6	36 (22.64%)
VAS 7–10	4 (2.52%)
**PBI (n, %)**	
0	82 (51.57%)
1–20	55 (34.59%)
21–50	19 (11.95%)
51–100	2 (1.26%)
> 100	1 (0.63%)

*CPSP* chronic post-surgical pain, *VAS* visual analogue scale, *PBI* Pain Burden Index.

Univariate analysis of all the patient characteristics was performed to identify variables associated with the development of CPSP. The factors identified as having an increased risk of CPSP included severe acute PP, a history of preoperative chronic pain, and psychological disorders (Table 3). As well as those previously acknowledged risk factors, several other rarely evaluated factors were analyzed, including whether a biological matrix was used, the size of the implant, postoperative complications, level of satisfaction with the reconstructed breasts, and whether the patient regretted having single-stage IBBR (Table 3). It was found that the patients with lower SS tended to develop more CPSP (Table 3). Although there was no statistical significance (p = 0.091, Table 3), seven out of nine patients who received bilateral single-stage IBBR developed CPSP.

**Table 3:** The univariate analysis of risk factors for CPSP.

Variables	CPSP	No CPSP	Odds ratio (95% CI)	p value
Age (years)	40.7 ± 6.4	41.4 ± 7.5	0.99 (0.95–1.04)	0.560
**Type of surgery**				0.104
With SLNB	55	53	0.84 (0.69–1.03)	
With ALND	15	27	1.58 (0.92–2.71)	
**Biological matrix**				1.000
Yes	62	65	0.98 (0.84–1.15)	
No	15	17	1.06 (0.57–1.98)	
**Unilateral or bilateral**				0.091
Unilateral	70	80	1.07 (0.99–1.16)	
Bilateral	7	2	0.27 (0.06–1.25)	
**Psychological problems**				0.027
Yes	44	32	0.68 (0.49–0.95)	
No	33	50	1.42 (1.04–1.94)	
**History of preoperative chronic pain**				0.002
Yes	25	10	0.38 (0.19–0.73)	
No	52	72	1.30 (1.09–1.55)	
**Acute PP**				0.004
Non-severe	53	72	1.28 (1.08–1.51)	
Severe	24	10	0.39 (0.20–0.76)	
**Postoperative complications**				0.392
Yes	8	5	0.59 (0.20–1.72)	
No	69	77	1.05 (0.95–1.15)	
**Regretted having single-stage IBBR**				0.034
Yes	12	4	0.31 (0.11–0.93)	
No	65	78	1.13 (1.01–1.26)	
The size of implant	222.4 ± 51.8	232.7 ± 57.9	1.0 (0.99–1.0)	0.238
SS	11.0 ± 2.7	12.5 ± 2.7	0.8 (0.71–0.91)	0.001

*CPSP* chronic post-surgical pain, *ALND* axillary lymph node dissection, *SNLB* sentinel lymph node biopsy, *PP* postoperative pain, *IBBR* implant-based breast reconstruction, *SS* satisfaction score.

The significant variables from the univariate analysis were included in the multivariate analysis (Table 4). Severe acute PP, a history of preoperative chronic pain, and a lower SS were all independently associated with the development of CPSP.

**Table 4:** The multivariate analysis of risk factors for CPSP.

Variables	Odds ratio (95% CI)	p value
Psychological disorders	1.70 (0.85–3.40)	0.135
History of preoperative chronic pain	3.39 (1.42–8.10)	0.006
Severe acute PP	2.80 (1.16–6.79)	0.023
SS	0.86 (0.75–0.99)	0.034
Regretted having single-stage IBBR	3.20 (0.90–11.43)	0.073

*PP* postoperative pain, *IBBR* implant-based breast reconstruction, *SS* satisfaction score.

**Figure 1:**
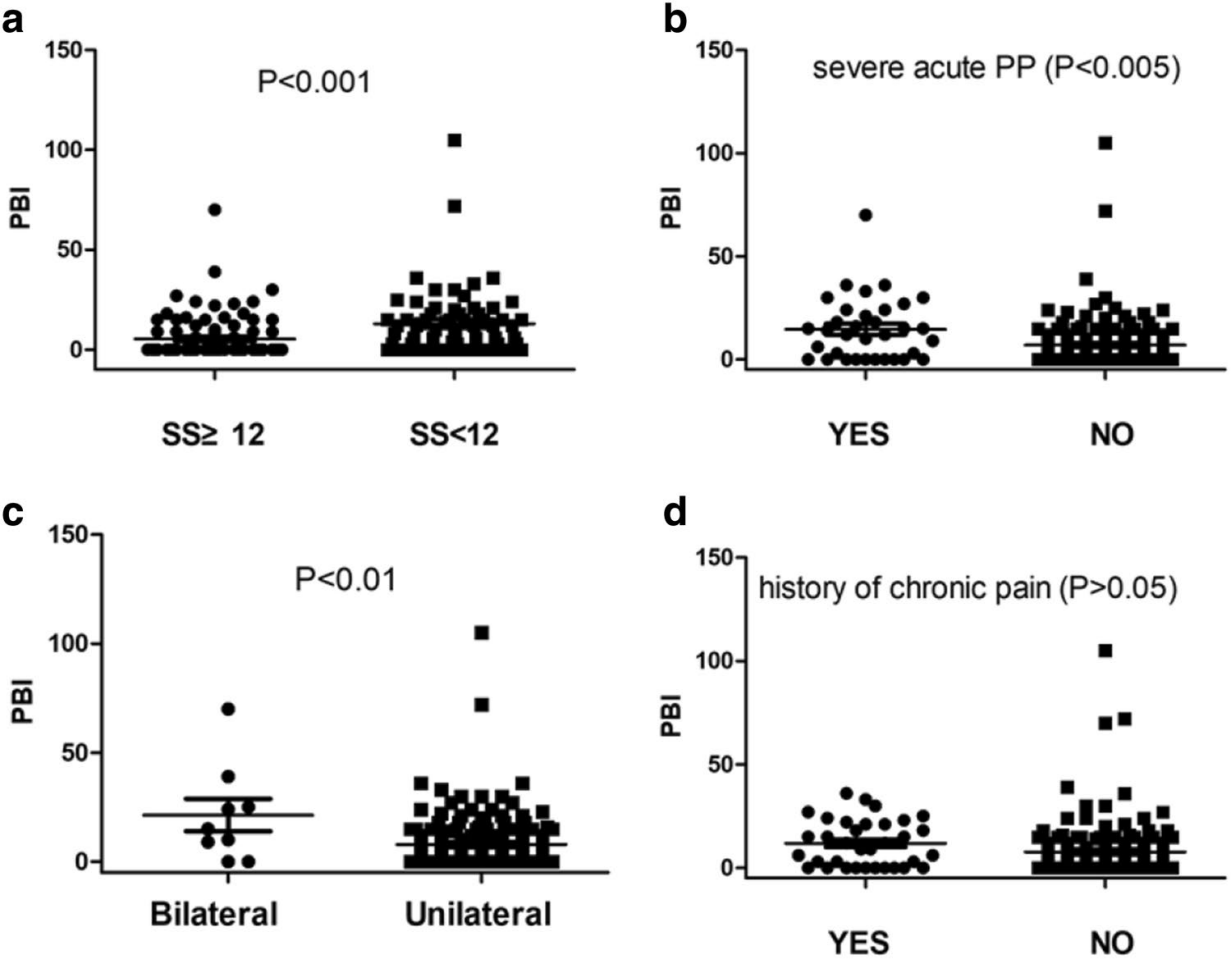
PBI of the patients in different subgroups. (**a**) the PBI for patients in the SS ≥ 12 group was significantly lower than that of patients in the SS < 12 group (p < 0.001); (**b**) the PBI for patients in the severe acute PP group was significantly higher than that of patients in the non-severe group (p < 0.005); (**c**) the PBI for patients receiving bilateral IBBR was significantly higher than that of patients receiving the unilateral procedure (p < 0.01); and (**d**) subgroup analysis showed that the PBI was similar for patients with or without a history of preoperative chronic pain (p > 0.05).

In the subgroup analysis, the patients were divided into the SS ≥ 12 vs. SS < 12 groups, unilateral vs bilateral groups, severe acute PP vs non-severe acute PP groups, and a history of chronic pain vs no history of chronic pain groups. The PBI of all the subgroups was compared, and the data is presented in Fig. 1.

## Discussion

This study showed that 48.43% of the patients who received single-stage IBBR following mastectomy developed CPSP and 2.52% were severe cases. Moreover, the patients who had lower SS developed more CPSP. Severe acute PP, a history of chronic preoperative pain, regretted having single-stage IBBR, and psychological disorders were also proven to be risk factors of CPSP, while lower SS, bilateral procedures, and severe acute PP were predictors of higher PBI.

Wink et al. found that, according to the National Surgical Quality Improvement Program database, the overall incidence of complications in 1,612 one-stage alloplastic reconstructions was 9%, whereas the incidence was 8.2% in this study. As the average age of patients with breast cancer is falling in China, more patients who have no chance of breast-conserving surgery are likely to consider single-stage IBBR. However, the incidence of CPSP after surgery has not been well-researched yet. At the same time, there is not enough awareness of the potential for treating CPSP, which means that the quality of life of some patients with CPSP is being unnecessarily neglected. There were 2.52% severe cases of CPSP (VAS ≥ 7) in this study, which was similar to the 2.1% reported by Roth^7^. However, even in severe cases, no one in this study got treatment for pain because they considered that it was inevitable following breast cancer surgery, so they simply tolerated it.

This study assessed the risk factors of CPSP following single-stage IBBR, including a history of chronic preoperative pain, acute PP, BMI, and age, as well as factors that were rarely investigated previously, namely SS, postoperative complications, the size of the implant, whether a biological matrix was used, and whether patients regretted having the IBBR. Table 5 summarizes the data of six published studies concerning the topic of CPSP after single-stage IBBR. Most of these studies contained a very small sample size, and some of them calculated the incidence of CPSP after reconstruction with a tissue expander or an implant indiscriminately^8,9^. This is likely to have led to a degree of inaccuracy because reconstructing with a tissue expander or an implant involves two different kinds of procedures, whereas this study enrolled 159 patients who all received single-stage IBBR. Moreover, all the cases were recruited within 1 year, and their operations were performed by the same specialized surgical team, which means that the changes in operational methods and differences in operational skills were comparatively few.

**Table 5:** Summary of methodological parameters for studies investigating CPSP following single-stage IBBR.

Study	Period (years)	Study design	Sample size of SSIBBR	Pain metric	Preop pain (Y/N)	SS (Y/N)	Biological matrix (Y/N)	Size of implant (Y/N)	Postoperative complications (Y/N)	Time since surgery (mths)	Prevalence of CPSP (%)
Hickey^10^	6	Retrospective	< 9	MPQ, VAS	N	N	N	N	N	–	43%
De Liveira^4^	5	Retrospective	< 68?	BPI, McGill	Y	N	N	N	N	6	38%
Weichman^9^	5	Prospective	94	MPQ-SF, NPRS	Y	N	N	N	N	3	?
Legeby^8^	1	Prospective	32	VAS	N	N	N	N	N	36	25%
Spivey^11^	3	Prospective	1	PBI	N	N	N	N	N	6	–
Henderson^12^	2	Retrospective	34	VAS	N	N	N	N	N	19	19%
This study	1	Retrospective	159	PBI/VAS	Y	Y	Y	Y	Y	12	48%

*MPQ-SF* McGill pain questionnaire-short form, *VAS* visual analog scale, *NPRS* numerical pain rating scale, *MPQ* McGill pain questionnaire, *BPI* brief pain inventory, *PBI* pain burden index, *SSIBBR* single-stage implant-based breast reconstruction, *SS* satisfaction score, *CPSP* chronic postsurgical pain.

Consistent with previous studies^2,13^, CPSP was proven to be significantly related to a history of chronic preoperative pain, psychological disorders, and severe acute PP, while lower SS, and regret for having had the IBBR were also found to be connected with higher incidence of CPSP. This result is a reminder to surgeons and anesthesiologists that it is important to communicate adequately with patients and let them make decisions after full consideration, and that it is essential that they not only understand the risk of developing CPSP after surgery, but they are also aware that it can be treated. In addition, increasing the patient’s SS with the reconstructed breast might help control CPSP. This would be a different approach from the situation at present, where CPSP management has substantially contributed to the current opioid crisis^14^. In this study, none of the patients had ever sought treatment, even for severe pain, which indicates that the issue of CPSP after breast cancer surgery has greatly been ignored by patients and healthcare workers alike in China. It is the responsibility of surgeons and anesthesiologists to educate patients and inform them that CPSP is a problem that can and should be treated.

How to explain the phenomenon that the lower SS was associated with a higher incidence of CPSP? Firstly, low SS might be associated with a lack of sensation and physical feeling and poor sexual and poor physical well-being, which could negatively influence self-confidence and self-identity, and meanwhile increase the incidence of CPSP. Secondly, patients had different cultural and educational backgrounds, which means they had different cognitive levels. Those who had more positive cognition tended to achieve higher SS and had fewer incidences of CPSP^15^. Or thirdly, patients with lower SS developed more psychological distress caused by an unexpected appearance, which predisposed them to develop CPSP more easily^16^.

For the treatment of CPSP, the development of transitional pain services, which identifies patients at risk and treats them early in a multidisciplinary approach, is suggested^17,18^. Although it has been reported that regional anesthesia (paravertebral block) did not reduce the frequency and severity of CPSP in breast cancer patients^19^, regional anesthesia technique is still a feasible option for CPSP treatment. Medicines, including opioids, non-steroidal anti-inflammatory drugs, anti-depressants, anti-epileptics, etc., might be effective. Physical therapies such as massage and thermal therapy might help. However, further research is warranted to study the prevention and treatment of CPSP in the future. Recently, the study by Caputo et al. reported that prepectoral implant positioning improved the level of SS with breast appearance and reduced the intensity of immediate PP, which provides patients willing to receive IBBR an alternative for CPSP prevention^20^. Regional nerve blocks such as intercostal nerve blocks were reported to be an effective way to control immediate PP^21,22^, as severe PP was one of the risk factors of developing CPSP, so reginal nerve blocks might have potential effects in CPSP prevention.

This study does have several limitations. First, it is a single center retrospective study with recall bias. Data collection was limited to the information present in the electronic medical records. The number of patients undergoing bilateral single-stage IBBR was too small for meaningful statistical analysis. However, seven out of nine patients who received bilateral single-stage IBBR developed CPSP, which suggested that bilateral IBBR might be one of its risk factors, which needs further elucidation in the future. Performing prophylactic single-stage IBBR following a mastectomy is not advisable, because CPSP might become a serious problem. Second, in our hospital, there is a specialized surgical team responsible for performing breast reconstruction surgery. In this study, we have enrolled only the patients from this surgical team to minimize surgical technique related bias. Therefore, we conducted a single-arm cohort study, and no control patients receiving mastectomy without immediate breast reconstruction were included.

In conclusion, this study demonstrated a significant incidence of CPSP following single-stage IBBR. It was possible to identify patient-specific characteristics associated with an increased risk of CPSP. Patients who have a lower SS developed more CPSP, and it is clear that lower SS, bilateral procedures, and severe acute PP are predictors of higher PBI. Although nearly half of the patients in this study experienced CPSP, none of them had ever pursued treatment. Both anesthesiologists and surgeons should be encouraged to counsel patients on the course and risks of CPSP following single-stage IBBR, and they should be made more aware of the potential need to solicit aggressive CPSP management for symptomatic women.

## Data Availability

All the data used and analyzed are available from corresponding authors upon the reasonable request.
